# Progress in Genito-Urinary Surgery

**Published:** 1902-05-17

**Authors:** 


### PROGRESS IN GENITO-URINARY SURGERY.

(*Continued from page* 100.)

**Urinary Fever.**—G. Buckstone Browne[6] in the Harveian Lectures discussed this subject. He believes this fever to be at its outset purely a suppression of urine, varying from a merely transitory condition to one of absolute and complete suppression; and that it is due to inhibition of the action of the kidney from urethral shock. The nerve supply of the urethra is very free. Many surgeons of experience, the writer says, will be able to recall some case of an elderly man who had a catheter passed and subsequently never secreted another drop of urine and died. The value of sedatives in the prevention of urinary fever is alluded to. Women and children never suffer from this form of fever. They have a urethra which is a urinary tract only, unlike that of the male which has a sexual function. The French are more susceptible to urinary fever than the Germans, and the Irish than the English. The more susceptible people are those who are the more nervous. If the suppression of urine is complete or considerable the kidney becomes engorged with blood and inflammation ensues and even suppuration may follow. The writer thinks, therefore, that although urinary fever may end in septicism, it is not primarily a blood poisoning.

**The Treatment of Rupture of the Bladder and Urethra.**—C. J. Bond[8] has contributed a paper on this subject. Of the former, the bladder injuries, his experience has been chiefly in cases of accidents in the hunting field, in which the horse has rolled over the rider and crushed the pelvis. There is a tender, board-like, dull area above the pubes spreading to the groins, and inability to empty the bladder. In all his cases in which the bladder was torn the rent was in its anterior wall, and due, he believes, to the direct puncturing of the vesical wall by the fractured bones. Hence the pre-vesical space should be opened first in these cases and the rent looked for. The peritoneum need not be opened unless injured. The urethra appears to be injured in the membranous portion through bilateral crushing of the pelvis, which drives the fractured sides of the pubic arch together like the blades of a pair of scissors. Where the pelvis is crushed antero-posteriorly the urethra is more likely to escape. In the commoner rupture of the urethra—the result, for instance, of a fall astride a beam—the soft urethra is crushed against the pubic arch, and the anterior portion is torn away from the more fixed proximal end. The rupture is then further forward than in fractured pelvis, and the separation of the torn ends may be considerable, *e.g.*, one or two inches. If in suprapubic exploration the rent is found to be sufficiently within reach, it should be carefully closed in the usual way with sutures. If it extends into the prostate suturing will probably fail, and efficient drainage must be secured. The writer advocates in such cases combined supra- and infra-pubic drainage, and accomplishes this by passing some such instrument as a sound, guided by the finger, through the rent in the anterior wall and through the internal meatus and prostatic urethra until it presents beneath the skin of the perineum over the membranous urethra, by cutting down upon it there, and by drawing a tube with the aid of the instrument from the perineal wound to the supra pubic one. The drainage holes in the tube are placed just within the neck of the bladder, and a small collar on the tube resting on the internal meatus keeps these holes in proper position. In the ordinary form of ruptured urethra from direct violence without fracture of the pelvis, Bond strongly advises that, where possible, the urine should be conducted externally behind the sutured portion, and that no catheter or foreign body should be left traversing the sutured urethra. When the supra-pubic drain is renewed, the author finds it an efficient way of keeping the patient dry to use an ice-bag with part of the front cut away, and with a piece of flanged rubber tubing passed through the back and into the bladder. Wool is then placed in this rubber receptacle and needs to be changed only twice during the night. In draining the supra-pubic space tubes may be brought out under one or both pubic rami by cutting on a sound passed from above. In ordinary ruptured urethra with separation of the two parts the difficulty due to the separation is best overcome by a free detachment of the distal spongy portion, together with the supporting corpora cavernosa from the front of the pubes and the partial separation of the suspensory ligament of the penis.

**The Treatment of Enlarged Prostate.**—Wallace[9] has considered this question. He stated that a patient might suffer from all the symptoms of prostatic hypertrophy and yet the gland might not be enlarged. A consideration of symptoms alone could not determine whether the prostate was enlarged. Enlargement of the gland detected by rectal examination did not of necessity imply mechanical obstruction; and, on the other hand, a small prostate might possess an obstructing intra-vesical enlargement. These facts afforded a plea for supra-pubic cystotomy. Whatever was the origin of the malady, it was often due to the formation of fibro adenomatous or fibro-myomatous tumours in the gland. Three varieties of enlargement might be recognised: (1) vascular, (2) fibrotic, and (3) fibro-adenomatous or fibro-myomatous. Any one of these might produce the symptoms of "chronic incomplete retention." Four methods of treatment were at present in use—(1) catheterism, (2) cystotomy, (3) castration or vasectomy, and (4) prostatectomy. Personally Wallace thought catheterism should be used if possible only for these cases where atony of the bladder had not been reached. The mortality of castration was as high as that of prostatectomy, if all cases were considered. The favourable cases for supra-pubic cystotomy were those in which there was a removable middle lobe or there were fibro-adenomatous tumours present. The writer did not believe that all prostates were suitable for even partial removal, and he believed that complete removal of the prostate without opening the urethra was an anatomical impossibility. He considered that supra-pubic cystotomy *per se* was not dangerous. H. L. Barnard[10] relates a case in which he enucleated prostatic adenomata successfully in a man of 62. After opening the bladder above the pubes the two lateral lobes of the prostate were felt projecting into the bladder as rounded egg-shaped masses, while between them posteriorly was a pedunculated middle lobe the size of a walnut. The mucous membrane of the floor of the bladder was incised over the right lobe for about one inch, and both lobes of the gland were gradually shelled out through this opening. The middle lobe came away with the right lateral one. There was considerable hæmorrhage, and at the conclusion the bladder was irrigated with equal parts of hazelene and water. The results were that at the end of three weeks the patient could hold his urine four hours and pass a good stream without delay. Barnard states that he followed P. J. Fryer in the method of operating. The patient lost from 15 to 20 ounces of blood on the table, and 10 ounces the following night. Barnard believes that in these operations the adenomatous tissue of the prostate is enucleated from the muscular and fibrous capsule and stroma, leaving the urethra and even the ejaculatory ducts intact, very similarly to the way in which adenomata of the thyroid are enucleated. In a fibrous enlargement the writer found it quite impossible to enucleate the gland, and all he could do was to cut away the obstructing portion. Barnard recommends the use of acid sodium phosphate in order to render urine acid, and states that it is far more efficacious than boric acid or ammonium benzoate. It is best administered in the strength of 1 drachm to a pint of water, flavoured according to taste, that quantity being taken daily as a beverage. It is specially efficacious when combined with urotropine.

**Bilocular Intrapelvic and Scrotal Hydrocele.**—J. Lacy Firth[11] records a case of this somewhat rare condition. The case was that of a labouring man aged 20, who complained of a swelling in the left side of the scrotum. The swelling had existed from infancy, and on one occasion had been tapped and fluid drawn off. The swelling was translucent, and had a very marked impulse on coughing. It could be almost completely reduced through the inguinal canal, like a hernia, but quickly returned when external pressure was removed. When one hand was laid on the abdomen and the other on the scrotum fluctuation was easily obtained, and similarly a thrill was easily transmitted from one part to the other on gently tapping either. A sudden pressure on any part of the abdomen immediately produced a visible expansion of the scrotal swelling. Both flanks were resonant on percussion. Fluctuation could not be detected by rectal examination. On opening the sac at an operation it was found that the forefinger could easily be passed from the scrotal sac through the inguinal canal into a large cystic cavity lying in the iliac fossa, this sac being extra-peritoneal. The testicle lay in the scrotal sac. The latter was cut across near the upper end of the testicle, and the upper part dissected up to the inguinal canal. With a little further dissection and gentle traction the whole of the intra-abdominal sac was removed. The writer points out that bilocular hydroceles of this type probably arise from faulty obliteration of the funiculo-vaginal process. In the so-called infantile hydrocele obliteration of this process has occurred near the internal abdominal ring, the rest of the process below that, including the tunica vaginalis, being distended with fluid. In the bilocular cases there is a similar scrotal sac, but in addition a second sac continuous with the other lying in the abdominal cavity. This suggests that a higher unobliterated portion of the funiculo-vaginal process has become distended with fluid. Macewen believes the abdominal sac is due to the funicular process existing as a sheath along the anterior aspect of the pelvic portion of the vas deferens, and being shut off from the peritoneal cavity at the deepest part of the vas, so that the process is continuous and patent from the testicle to the pelvis. If such a tube became distended with fluid it would give rise to bilocular hydrocele of the type under consideration. Reference to the literature on the subject shows that 25 cases have been reported, including the present one.

[6] Lancet, Nov. 16, 1901, p. 1317. [8] Lancet, Nov. 23, 1901, p. 1404. [9] Lancet, Dec. 14, 1901, p. 1676. [10] Lancet, Nov. 9, 1901, p. 1264. [11] B. M. J., Nov. 16, 1901, p. 1463.

